# Supplementary material for: Sustainable heavy metal immobilization in contaminated soils using plant-derived urease-driven biomineralization
Source: PLoS One. 2025 Sep 19;20(9):e0331241. doi: 10.1371/journal.pone.0331241 (PMC12449025; doi:10.1371/journal.pone.0331241)
Supplement: Attachment — Submitted filename: PONE-D-25-18160.docx [file pone.0331241.s002.docx]

The manuscript investigates the immobilization of heavy metals by a plant-derived urease-driven carbonate precipitation process, providing a new idea for the remediation of heavy metal-contaminated soils. After careful review, the work was found to be innovative, the analysis of the data results was rather lacking, and major revisions were needed. Specific comments are as follows:

**Abstract**

Does the “structural resilience” of the enzyme have an effect on the rate of heavy metals fixation? No supporting data seems to be found in the article.

**Introduction**

1. Lines 48-51: The MICP process of ureolytic bacteria does not necessarily require strict aseptic control. The MICP process can also be carried out by ureolytic bacteria as the main group of bacteria, and there have been academic papers on the subject. On the other hand, MICP technology has now developed relevant bacterial agents, and the cost has been reduced to a certain extent. It is suggested to modify the related expressions.

2. Lines 64-67: the study by Wang et al. uses what appears to be mercapto-palygorskite for Cd immobilization and does not involve the EICP technique, please check that the reference is cited correctly.

3. The introduction of MICP and EICP is relatively simple, and it is recommended that a literature review section be added to make the reader more aware of these two techniques and to improve the readability of the article.

**Materials and Methods**

1. For the selection of some methods, it is recommended to supplement the relevant reference standards or literature, such as the pH of the enzyme extraction process species, Tisser's method for morphological extraction, etc.

2. Missing tables for orthogonal experiments.

**Results**

1. Lines 155, 163, and 166, Canavalia ensiformis and Citrullus lanatus, C. ensiformis are not mentioned in the previous section or Fig. 1, and it is suggested that the names be modified to Soyben , Jack Bean, and Watermelon to harmonize the names.

2. Lines 158-160, This enhanced performance was attributed to its superior urea hydrolysis rate of 15.8 μmol NH₃/min/mg protein, which generated 40% more carbonate ions compared to soybean urease under identical conditions. This is not reflected in Fig.1. and no references are cited.

3. Lines 166-169, suggest citing relevant literature as support.

4. In Fig. 2, it is suggested that all three extracted enzymes are compared with microbial urease for tolerance.

5. Lines 181-187 lack relevant graphical data support or relevant literature citations.

6. Lines 192-195, after the action of Jack bean, it should be that the soluble cadmium concentration is 1.8 mg/kg, not reduced from 6.5 mg/kg to 1.8 mg/kg. There are similar problems in the next few expressions, so please check them and revise them all.

7. Lines 200-201, “These findings suggest preferential carbonate precipitation mechanisms favoring lead and zinc ions over cadmium.” Should comparisons be made between different initial lead, cadmium, and zinc contamination concentrations in terms of removal, thus illustrating the effect of immobilization. When the initial concentration is large, the fixation rate may not be high, but the amount of fixation is high.

8. Lines 203-205, “Watermelon seed urease increased zinc fixation efficiency from 76.4% under single-metal conditions to 83.2% in tri-metallic systems, reducing soluble zinc concentrations from 3.1 mg/kg to 2.6 mg/kg.” This seems inconsistent with the data in Fig. 3, where the fixation efficiency appears to be just under 70% in the Cd+Pb+Zn contaminated system, not 83.2% as expressed. In addition, which heavy metal does the soluble metal concentration and immobilization efficiency refer to in the Cd+Pb+Zn pollution system, or the total amount? Please check the data in Fig. 3 for problems or modify Fig. 3 to show the remaining heavy metal concentration as well as the immobilization efficiency more clearly.

9. Lines 207-211, the relevant statements are missing data support such as pictures and tables.

10. Fig. 6 needs further adjustments to improve clarity and readability, and it is recommended that the plotting range be reduced to 20-50°.

11. Lines 271-286, lack a figure or table for intensity reduction, and the XRD data do not have a corresponding figure.

12. In Table 1, what rules, or what basis, are used for the relevant costing, and please give appropriate support.

**Discussion**

1. Lines 356-368, Fig. 3 does not demonstrate that systematic parameterization of the urea-CaCl_2_ stoichiometry mitigates the reduction in porosity caused by uncontrolled hydrolysis of urea, thus solving the key challenges of field implementation. The results of the experiments on orthogonal experiments as well as permeability are not represented in the manuscript.

2. Lines 369-380, demonstrate the superiority of plant-derived urease-driven MICP processes in terms of their ability to regulate pH, which should be compared to microbial-driven MICP processes in addition to lime stabilization.

3. Lines 402-404, XRD information on SiO_2_ is not reflected in the manuscript, please confirm if the information is provided.

4. Lines 425-426, Fig. 4 does not prove that the mass loss of surface carbonate reached 17.5%, e.g., some of the urease-induced formation of carbonate did not bind to the heavy metal ions, but only formed calcium carbonate, which may also be decomposed in the presence of acid.

5. Lines 430-436, “X-ray diffraction patterns indicate the selective dissolution of amorphous carbonate phases, as shown by the attenuation of the broad peak at 2θ = 20-30°, while crystalline cadmium carbonate domains (sharp peak at 24.9°) retain 82.5% of their initial mass (Fig. 5). ” There is no corresponding photographic data to support this conclusion.

6. Lines 446-449, “Phytocompatibility testing demonstrated the restoration of ecological functionality in treated soils, as evidenced by Lactuca sativa achieving 85-92% germination efficiency, which is comparable to non-contaminated reference samples (95% germination)”. There are no relevant experimental results to support the data.

7. Lines 458-465, the results of TCLP leaching are not shown in the manuscript. The results of the assay for urease activity are also not shown in the manuscript.

8. lines 470-476, the penetration rate of the study is not shown in the manuscript.

9. Lines 477-481, data on the treatment effects of this treatment technique in different soils, are also not represented accordingly in the manuscript.

10. Lines 508-510, “Simultaneously, constructing machine learning-enhanced soil compatibility frameworks by systematically analyzing over 10,000 global soil profiles could facilitate centralized databases that elucidate region-specific urease-soil interactions [37].” Doesn't seem to be relevant to your research.

**Conclusion**

It is recommended that the conclusion section be streamlined and that the most central conclusion of the elaboration section be sufficient.
