# Supplementary material for: Sustainable heavy metal immobilization in contaminated soils using plant-derived urease-driven biomineralization
Source: PLoS One. 2025 Sep 19;20(9):e0331241. doi: 10.1371/journal.pone.0331241 (PMC12449025; doi:10.1371/journal.pone.0331241)
Supplement: Attachment — Submitted filename: Response letter.docx [file pone.0331241.s004.docx]

**Sustainable Heavy Metal Immobilization in Contaminated Soils Using Plant-Derived Urease- Driven Biomineralization**

**Response letter to Reviewers’ comments**

**Comments from the Editor：**

1. Please ensure that your manuscript meets PLOS ONE's style requirements, including those for file naming.

Response：We thank the editor for the reminder regarding PLOS ONE's formatting and file naming requirements. We have reviewed all submission files to ensure full compliance with the journal's formatting guidelines, including figure captions, reference formatting, section organization, and file naming conventions. All necessary adjustments have been made accordingly in the revised submission.

2. In your Methods section, please provide additional information regarding the permits you obtained for the work. Please ensure you have included the full name of the authority that approved the field site access and, if no permits were required, a brief statement explaining why.

Response：We appreciate the editor’s reminder regarding fieldwork permits. In response, we have revised the Methods section to clarify that the soil samples were collected from publicly accessible, abandoned industrial sites that are neither protected nor privately owned. No specific permissions were required for soil collection, and all activities were conducted in compliance with local institutional and environmental regulations. The corresponding statement has now been added to the “Soil Sampling and Characterization” section.

3. Thank you for stating the following in the Acknowledgments Section of your manuscript:……Please remove any funding-related text from the manuscript and let us know how you would like to update your Funding Statement. Please include your amended statements within your cover letter; we will change the online submission form on your behalf.

Response：We thank the editor for highlighting the discrepancy regarding the placement of funding information. As instructed, we have removed all funding-related content from the Acknowledgments section of the manuscript.

4. We note that your Data Availability Statement is currently as follows: All relevant data are within the manuscript and its Supporting Information files. Please confirm at this time whether or not your submission contains all raw data required to replicate the results of your study. Authors must share the “minimal data set” for their submission. PLOS defines the minimal data set to consist of the data required to replicate all study findings reported in the article, as well as related metadata and methods

Response：We confirm that our submission contains all raw data required to replicate the results of the study. All relevant datasets—including numerical values underlying graphs, statistical analysis results, and extracted data points used in Figures and Tables—have been provided in the Supporting Information files accompanying the manuscript. We have reviewed the Data Availability Statement and confirm its accuracy. No ethical, legal, or institutional restrictions apply to the sharing of this dataset.

5. Please amend either the abstract on the online submission form (via Edit Submission) or the abstract in the manuscript so that they are identical.

Response：We thank the editor for pointing this out. We have carefully reviewed the abstract in both the manuscript and the online submission system, and we have now made them identical to ensure consistency. The abstract in the manuscript was revised to match the version submitted online.

6. We note that one or more reviewers has recommended that you cite specific previously published works. As always, we recommend that you please review and evaluate the requested works to determine whether they are relevant and should be cited. It is not a requirement to cite these works. We appreciate your attention to this request.

Response：We appreciate the editor's reminder and have carefully reviewed all references suggested by the reviewers. Citations were added where relevant and scientifically appropriate to acknowledge prior contributions and provide context. Where suggested works were not cited, we respectfully determined that they were not directly pertinent to the scope or findings of the current study.

Another point that requires clarification is that we observed a total of 17 review comments from Reviewer 1 in the email. Upon carefully addressing the comments, we noticed that comments 9–13, as marked, constituted a single review comment. Due to its length, this comment may have been misnumbered automatically during formatting or line breaks. As a result, Reviewer 1 effectively provided 13 distinct comments, each of which we have addressed individually.

**Comments from Reviewer#1:**

Some of the draw backs which needs justification are listed below

1. Limited Scope of Heavy Metals: The study focuses only on cadmium, lead, and zinc. The effectiveness of this EICP system on other heavy metal contaminants is unknown and requires further investigation.

Response：We appreciate the reviewer’s insightful observation. The current study indeed focuses on cadmium (Cd²⁺), lead (Pb²⁺), and zinc (Zn²⁺) due to their prevalence in typical nonferrous smelting sites and their high mobility in soil environments. However, we fully recognize the necessity of assessing the broader applicability of the EICP system to other heavy metal contaminants, such as arsenic (As), nickel (Ni), and chromium (Cr), which commonly co-occur in post-industrial soils. To address this limitation, we have now included a dedicated paragraph in the “Limitations and Future Directions” section that explicitly acknowledges this gap and outlines plans for future studies involving these additional contaminants. This addition enhances the comprehensiveness and transparency of our research. (Line 607-619)

2. Enzyme Dosage Optimization: While the study optimized enzyme dosage, the process of determining the optimal 1.2 U per gram of soil might be sensitive to variations in soil type and contamination levels, potentially requiring site-specific adjustments.

Response：We extend our gratitude to the reviewer for this insightful observation. We concur entirely that enzyme dosage optimization is inherently contingent upon the heterogeneity of soil physicochemical properties and varying degrees of heavy metal contamination. Indeed, this represents a critical factor in practical field applications. To elucidate this point, we have refined the relevant section in the Discussion to explicitly state that the reported optimal dosage (1.2 U/g soil) pertains specifically to the soil textures, pH levels, and contaminant concentrations examined in our trials. Furthermore, we underscore the necessity for future applications to integrate localized pilot testing to adjust enzymatic input according to site-specific conditions. This enhancement not only improves the clarity of our findings but also strengthens their generalizability. (Line 371-380)

3. Potential for Scalability Challenges: The abstract doesn't discuss the feasibility and cost-effectiveness of scaling up this EICP system for large-scale field applications. Transitioning from lab experiments to real-world implementation can present significant engineering and logistical challenges.

Response：We sincerely appreciate the reviewer’s suggestion to incorporate a discussion of the scalability and cost-effectiveness of the EICP system into the abstract. Although the main text of the manuscript, especially in the "Field-Scale Validation" and "Alignment with National Environmental Policies" sections, already delves deeply into implementation feasibility, we concur that emphasizing these aspects in the abstract would enhance clarity and underscore the practical significance of our research. Consequently, we have revised the abstract to include a concise statement regarding cost-effectiveness and scalability, supported by field validation results. This adjustment provides a more comprehensive summary, balancing scientific innovation with real-world applicability. (Line 29-32)

4. Long-Term Stability Beyond Tested Conditions: While durability under simulated acid rain and freeze-thaw was assessed, the long-term stability of the immobilized heavy metals over decades and under other environmental stressors (e.g., changes in groundwater chemistry, biological activity) remains to be fully evaluated.

Response：We sincerely appreciate the reviewer for pointing out this critical limitation. Indeed, although our study assessed the short- to medium-term durability of EICP-treated soils under acid rain and freeze-thaw cycles, we concur that predicting their long-term performance over decadal timescales under more complex environmental conditions—such as fluctuations in groundwater chemistry, microbial activity, or redox potential—remains challenging and warrants further investigation. To address this concern, we have included a dedicated paragraph in the “Limitations and Future Directions” section explicitly recognizing these knowledge gaps. Additionally, we propose future research avenues, such as long-term field monitoring and coupled biogeochemical modeling, to evaluate the temporal evolution of carbonate phases and potential metal remobilization processes. (line 585-596)

5. Line 85 Please write the main objectives of your work and the research gap before concluding the introduction. please refer the following papers 10.1007/s40098-022-00638-8, 10.1007/s40098-022-00638-8, 10.3390/buildings14040909 to help you answer the comment.

Response：We sincerely appreciate the reviewer's valuable suggestion and the provision of relevant references that have guided our revision process. In response, we have meticulously revised the closing paragraph of the Introduction to clearly delineate the research gap and explicitly state the objectives of this study. Drawing inspiration from the structure of the referenced studies—which emphasize problem framing, scope clarification, and hypothesis-driven aims—we now highlight (i) the critical need for plant-derived urease systems capable of maintaining robust performance under co-contaminated, field-realistic conditions, and (ii) the significant gap in systematic evaluation regarding durability and process optimization under multi-metal competition scenarios. These revisions enhance the scientific rigor of the study and ensure alignment with best practices in problem-driven research reporting.

6. Line 143 why only Carbonate was sequentially extracted even though there are more fractions available since the data is available from your work. and please elaborate refer the following 10.1007/s40098-020-00464-w to answer this comment.

Response：We sincerely appreciate the reviewer's insightful comment. In EICP systems, the primary mechanism for heavy metal immobilization is the formation of stable metal carbonates, such as otavite (CdCO₃) and hydrocerussite [Pb₃(CO₃)₂(OH)₂]. Consequently, our study specifically targeted the carbonate-bound fraction to directly assess the efficacy of the biomineralization pathway under investigation. This methodological choice aligns with previous research (e.g., Ahmad et al., 2021, DOI: 10.1007/s40098-020-00464-w), which underscores the importance of conducting fraction-specific analyses that correspond to the predominant remediation mechanisms. Although we recognize that other fractions could offer valuable insights into long-term redistribution dynamics, the objective of this study was to evaluate the immediate immobilization efficiency driven by carbonate precipitation. A more comprehensive fractionation analysis, along with an evaluation of post-treatment mobility, will be addressed in subsequent studies aimed at understanding long-term environmental behavior.

7. List out the objectives of the present work in the last paragraph.

Response：We appreciate the reviewer’s suggestion to further clarify the research objectives. In response, we have revised the concluding paragraph of the Introduction to explicitly articulate the objectives of this study in a concise and logically structured manner. This adjustment enhances the clarity and coherence of the Introduction, aligning with standard practices in environmental and geotechnical engineering research.

8. Line 106-108 soybean (Glycine max) please explain glycine max.

Response：We appreciate the reviewer's suggestion to clarify the scientific name Glycine max. In response, we have revised the relevant sentence to briefly explain that Glycine max, commonly known as the soybean plant, is widely cultivated for its high protein content and seeds rich in urease. This clarification aims to enhance understanding for readers who may be less familiar with botanical nomenclature.

9. The mixture was subsequently clarified via centrifugation at 10,000 ×g for 15 minutes. Comparable extraction protocols were applied to soybean (Glycine max) and watermelon seed (Citrullus lanatus) samples, utilizing pH-optimized buffering systems adjusted to 7.5 and 6.5, respectively, to ensure species-specific enzyme stability……Not very clear cannot comprehend whether you have extracted urease from soybean and water melon seeds I am not able to find data from these two ingredients in the paper.

Response：We thank the reviewer for pointing out the lack of clarity regarding urease extraction from soybean and watermelon seeds. We confirm that urease was indeed extracted from both plant sources using the same protocol applied to jack bean seeds, with species-specific buffer adjustments to optimize pH stability. The performance data for all three plant-derived ureases—including soybean and watermelon—are presented in Figures 1 and 3. To address this confusion, we have revised the Methods section to explicitly state that all three urease extracts were prepared and tested. We also clarified the source attribution for the data presented in the Results section.

10. Line 118 and 119, 1.2 U g⁻¹ soil, urea-CaCl₂ molar ratios systematically varied from equimolar (1:1) …….. The authors have determined 0.2 – 1.2 Urease per gram of soil as optimum dosage but it is not clear how to prepare samples so as to arrive at that dosage. Please give details in terms of how many grams of jack bean seed derived powder should be added to obtain 1.2 U.. of urease similarly for soybean and water melon seeds. Else this cannot be replicated.

Response：We appreciate the reviewer’s important observation regarding experimental reproducibility. In response, we have revised the “Plant Urease Extraction and Activity Assay” section to provide details on the specific activity (U/mg) of crude urease extracts from jack bean, soybean, and watermelon seeds. Based on this, we calculated the required mass of each extract needed to achieve the target dosage of 1.2 U per gram of soil. These clarifications ensure that the EICP treatment protocol can be replicated using plant-derived urease sources.

11. Fig 1. X axis is urease dosage in U/g but not clear how you have arrived at it, y axis “immobilization efficiency” please use a simple word as percentage retained or sorption efficiency.

Response：We thank the reviewer for the constructive suggestions regarding Figure 1. To improve clarity and replicability, we have revised the Methods section to explain how the urease dosage in U/g soil was calculated based on the specific enzymatic activities of each crude extract (now reported in U/mg), and how this was translated into the corresponding mass of enzyme powder added per gram of soil. Additionally, we have revised the y-axis label in Figure 1 to read “Metal Retention (%)” instead of “Immobilization Efficiency” to ensure the terminology is accessible and self-explanatory to a broad readership.

12. Table 1 economic analysis of soybean is not done.

Response：We thank the reviewer for pointing out the omission of soybean from the economic comparison in Table 1. We have now revised Table 1 to include the estimated production cost and treatment cost for soybean-derived urease. The calculation was based on current market prices of soybean seeds and the experimentally measured urease activity. Specifically, the production cost of crude soybean urease was estimated at 6.3 USD/kg, considering an average soybean market price of 5.5–6.5 USD/kg and an approximate extraction efficiency of 85%. The urease activity of soybean extract was measured as 9.4 U/mg, which corresponds to 9400 U/g. To achieve a dosage of 1.2 U/g soil, approximately 128 mg of crude extract is required per gram of soil. This translates to 0.128 kg extract per kg soil, resulting in an input cost of 0.81 USD/kg soil. Assuming a bulk density of 1.5 g/cm³, 1 cubic meter of soil contains approximately 1500 kg, yielding an estimated treatment cost of 59 USD/m³ for soybean-derived urease. This cost is slightly higher than jack bean or watermelon seed urease, but significantly lower than microbial MICP systems, making soybean a viable intermediate option in terms of economic feasibility. These values have now been added to Table 1 to complete the comparative analysis.

13. In the Conclusions part please provide the final outcome of this work in a sentence which shall be the punch line of this paper. Please rephrase the entire conclusion as it is non comprehensive and difficult to understand the outcomes.

Response：We appreciate the reviewer’s suggestion to improve the clarity and comprehensiveness of the Conclusions section. In response, we have completely rephrased this section to better summarize the study’s major findings, implications, and contributions. A concise punch line has also been added at the end to highlight the central outcome of the work.

**Comments from Reviewer#2:**

The manuscript investigates the immobilization of heavy metals by a plant-derived urease-driven carbonate precipitation process, providing a new idea for the remediation of heavy metal-contaminated soils. After careful review, the work was found to be innovative, the analysis of the data results was rather lacking, and major revisions were needed. Specific comments are as follows:

**Abstract**

Does the “structural resilience” of the enzyme have an effect on the rate of heavy metals fixation? No supporting data seems to be found in the article.

Response：We thank the reviewer for this insightful comment. In the original Abstract, we attributed the high immobilization efficiencies to “structural resilience and catalytic specificity.” While the catalytic specificity of different urease sources was experimentally validated, the term “structural resilience” may imply direct evidence of conformational or thermal stability, which was not explicitly assessed in this study. We agree that this wording may overstate the interpretation. To ensure scientific accuracy, we have revised this sentence in the Abstract to reflect the observed retention of enzymatic function under environmental stress, which is directly supported by the acid rain and freeze–thaw durability results.

**Introduction**

1. Lines 48-51: The MICP process of ureolytic bacteria does not necessarily require strict aseptic control. The MICP process can also be carried out by ureolytic bacteria as the main group of bacteria, and there have been academic papers on the subject. On the other hand, MICP technology has now developed relevant bacterial agents, and the cost has been reduced to a certain extent. It is suggested to modify the related expressions.

Response：We thank the reviewer for pointing out the need to revise our characterization of MICP technology. We agree that MICP no longer universally requires sterile conditions and that commercial bacterial agents and mixed microbial consortia have significantly improved operational flexibility and cost. To address this, we have revised the relevant sentence in the Introduction to more accurately reflect the current status of MICP development and its remaining limitations under variable field conditions.

2. Lines 64-67: the study by Wang et al. uses what appears to be mercapto-palygorskite for Cd immobilization and does not involve the EICP technique, please check that the reference is cited correctly.

Response：We sincerely thank the reviewer for pointing out the inconsistency in the citation. Upon re-evaluation, we acknowledge that the previously cited study by Wang et al. does not directly involve the EICP process and therefore should not be used to support the claim regarding heavy metal immobilization via EICP in calcareous versus acidic soils. To address this, we have replaced the reference with a more appropriate study by Moghal et al. (2020), titled “Heavy Metal Immobilization Studies and Enhancement in Geotechnical Properties of Cohesive Soils by EICP Technique” published in Applied Sciences. This study directly compares the performance of EICP treatment in acidic (red soil) and alkaline (black cotton soil) conditions and demonstrates that EICP protocols applied uniformly to different soil types result in variable cadmium immobilization efficiencies, with acidic soils showing a greater relative improvement. This substantiates our claim regarding the need for context-driven EICP design. The citation has been updated accordingly in the revised manuscript.

3. The introduction of MICP and EICP is relatively simple, and it is recommended that a literature review section be added to make the reader more aware of these two techniques and to improve the readability of the article.

Response：We thank the reviewer for the insightful suggestion. Rather than introducing a separate literature review section—which might disrupt the logical flow of the Introduction—we have carefully expanded the discussion of both MICP and EICP techniques within the existing structure. Specifically, we integrated recent literature published in the past few years to deepen readers' understanding of each method's mechanism, performance, and limitations within the context of heavy metal remediation. These revisions maintain the cohesion and focus of the Introduction while addressing the reviewer’s concern regarding contextual completeness.

**Materials and Methods**

1. For the selection of some methods, it is recommended to supplement the relevant reference standards or literature, such as the pH of the enzyme extraction process species, Tisser's method for morphological extraction, etc.

Response：We thank the reviewer for emphasizing the need for methodical transparency. In response, we have enriched the Materials and Methods section with rigorously validated references that justify key methodological choices:

(1) Extraction buffer pH: The selected pH values for urease extraction from jack bean, soybean, and watermelon seed (pH 8.0, 7.5, and 6.5, respectively) are based on the comparative study by Khodadadi Tirkolaei et al. (2020), which demonstrated optimal crude plant urease activity and carbonate precipitation efficiency under these conditions
(2) Enzyme extraction procedure: Our protocol for crude urease extraction (homogenization, filtration, centrifugation at 10,000×g for 15 min) follows the validated method described by Khodadadi Tirkolaei et al. (2020), ensuring reliable enzyme activity.
(3) Carbonate-bound metal fraction extraction: The quantification method is adapted from the sequential extraction protocol originally developed by Tessier et al. and reviewed in detail by Ali et al. (2024), which is recognized for accurate heavy metal fractionation in contaminated soils.

These additions enhance the clarity, reproducibility, and scientific rigor of our work.

2. Missing tables for orthogonal experiments.

Response：We thank the reviewer for this valuable suggestion. In response, we have added two supplementary tables to clarify the experimental structure:

(1) A single-factor optimization test on urease dosage (0.2, 0.5, 0.8, and 1.2 U/g soil), which corresponds to the data presented in Figure 1. This experiment was conducted under fixed conditions (urea:CaCl₂ = 1.5:1 and soil pH = 7.5).
(2) A comparative test on single-metal (Cd²⁺, Pb²⁺, Zn²⁺) versus multi-metal (Cd²⁺ + Pb²⁺ + Zn²⁺) systems, performed under the optimized EICP parameters. This design clarifies how contaminant interactions affect immobilization efficiency, while all other reaction parameters remained constant.

These tables have now been included in the supplementary section as Table 1 and Table 2. Both tables and corresponding details have been incorporated into the EICP Treatment Protocol section of the Materials and Methods, and are cited in the revised Results section.

**Results**

1. Lines 155, 163, and 166, Canavalia ensiformis and Citrullus lanatus, C. ensiformis are not mentioned in the previous section or Fig. 1, and it is suggested that the names be modified to Soyben , Jack Bean, and Watermelon to harmonize the names.

Response：Thank you for this helpful suggestion. In the revised manuscript, we have replaced the scientific names Canavalia ensiformis, Citrullus lanatus, and C. ensiformis in the Results and Discussion sections with their common names—jack bean, watermelon, and soybean—respectively, to ensure consistency and clarity throughout the text and figures. This modification harmonizes terminology across all sections and aligns with the nomenclature used in Figure 1.

2. Lines 158-160, This enhanced performance was attributed to its superior urea hydrolysis rate of 15.8 μmol NH₃/min/mg protein, which generated 40% more carbonate ions compared to soybean urease under identical conditions. This is not reflected in Fig.1. and no references are cited.

Response：We thank the reviewer for this important comment. We agree that the previous quantitative statement was unsupported by Figure 1 data and lacked appropriate references. We have revised the sentence to remove the unsupported quantitative claim and instead provide a qualitative description based on our observed enzyme activity trends. Additionally, we have added a citation to Liu et al. (2023), which reported that jack bean-derived crude urease demonstrates higher ureolytic activity than soybean-derived urease in EICP applications. This change has been implemented in the Results section, in the paragraph discussing comparative enzyme performance.

3. Lines 166-169, suggest citing relevant literature as support.

Response：Thank you for this insightful suggestion. We have now cited a 2022 study by Német et al., which experimentally demonstrates that Zn²⁺ ions (e.g., from the zinc-imidazole framework) inhibit urease activity and lead to enzyme conformational changes and complex formation, reducing catalytic performance. This citation has been added to the relevant sentence in the Results section.

4. In Fig. 2, it is suggested that all three extracted enzymes are compared with microbial urease for tolerance.

Response：Thank you for the helpful suggestion. We agree that including microbial urease as a reference group would improve the context of enzyme tolerance comparison. However, due to resource and scope limitations in this study, we did not experimentally assess microbial urease tolerance in parallel with the three plant-derived ureases. To address this, we have revised the Results section to clarify this limitation, and we have noted in the Discussion section that future work will include systematic comparisons with microbial urease under identical conditions to strengthen the conclusions.

5. Lines 181-187 lack relevant graphical data support or relevant literature citations.

Response：Thank you for this valuable comment. We agree that the original statement contained quantitative claims that were not supported by figure data or cited literature. To address this, we have revised the sentence to remove the specific numerical values and reframed it as a qualitative observation, referencing the known role of cysteine-rich motifs in metal tolerance of plant ureases as reported by Gautam (2023). The revised sentence avoids unsupported quantitative claims while preserving the discussion of structural differences potentially contributing to metal resistance.

6. Lines 192-195, after the action of Jack bean, it should be that the soluble cadmium concentration is 1.8 mg/kg, not reduced from 6.5 mg/kg to 1.8 mg/kg. There are similar problems in the next few expressions, so please check them and revise them all.

Response：We thank the reviewer for carefully identifying this issue. We agree with the reviewer’s observation and have revised the sentence to state directly the measured final soluble cadmium concentration after treatment, rather than implying a reduction from an initial value. We have thoroughly reviewed the manuscript and revised similar expressions to ensure consistency and scientific accuracy throughout the document. A total of four modifications were made to the text.

7. Lines 200-201, “These findings suggest preferential carbonate precipitation mechanisms favoring lead and zinc ions over cadmium.” Should comparisons be made between different initial lead, cadmium, and zinc contamination concentrations in terms of removal, thus illustrating the effect of immobilization. When the initial concentration is large, the fixation rate may not be high, but the amount of fixation is high.

Response：We appreciate the reviewer’s insightful suggestion. We agree that comparing both removal efficiencies and absolute amounts of heavy metal immobilization under differing initial concentrations would provide a more comprehensive understanding of the precipitation mechanism. While the current study focused on percentage immobilization efficiency, we have now added a note in the Discussion section emphasizing this point and highlighting that future work should systematically examine removal efficiencies versus absolute quantities immobilized, particularly when initial contamination levels differ between metals.

8. Lines 203-205, “Watermelon seed urease increased zinc fixation efficiency from 76.4% under single-metal conditions to 83.2% in tri-metallic systems, reducing soluble zinc concentrations from 3.1 mg/kg to 2.6 mg/kg.” This seems inconsistent with the data in Fig. 3, where the fixation efficiency appears to be just under 70% in the Cd+Pb+Zn contaminated system, not 83.2% as expressed. In addition, which heavy metal does the soluble metal concentration and immobilization efficiency refer to in the Cd+Pb+Zn pollution system, or the total amount? Please check the data in Fig. 3 for problems or modify Fig. 3 to show the remaining heavy metal concentration as well as the immobilization efficiency more clearly.

Response：We thank the reviewer for carefully noting this inconsistency. Upon reviewing our experimental records and Figure 3, we confirm that the zinc immobilization efficiency in the Cd+Pb+Zn co-contaminated system using watermelon seed urease is approximately 68%, not 83.2% as previously stated. We have corrected this in the Results section. Furthermore, we clarified that the values presented refer specifically to zinc in the Cd+Pb+Zn system, not the total heavy metal removal or average. Figure 3 already separately displays each metal’s residual concentration and immobilization efficiency, and we revised the text to explicitly state this and avoid confusion.

9. Lines 207-211, the relevant statements are missing data support such as pictures and tables.

Response：Thank you for this important comment. We carefully re-examined our dataset and found that the previously mentioned quantitative statement was not fully supported by the presented experimental results. Therefore, we have revised this sentence to remove the specific numerical claim and instead describe the observed trend qualitatively, emphasizing that parameter optimization improved lead immobilization performance under co-contamination conditions.

10. Fig. 6 needs further adjustments to improve clarity and readability, and it is recommended that the plotting range be reduced to 20-50°.

Response：We thank the reviewer for this helpful suggestion. Following your advice, we have revised Figure 6 by narrowing the plotting range to 20-50°, which covers the region containing all major characteristic peaks while improving clarity and readability.

11. Lines 271-286, lack a figure or table for intensity reduction, and the XRD data do not have a corresponding figure.

Response：We thank the reviewer for the valuable suggestion regarding the need for figures or tables presenting XRD intensity reduction and supporting the discussion of post-cycling mineralogical changes. We would like to clarify that this information is qualitatively presented in the manuscript. Specifically, Figure 6 shows the XRD patterns of EICP- and MICP-treated soils before and after freeze-thaw cycling, where differences in the retention of characteristic peaks corresponding to CdCO₃, Pb₃(CO₃)₂(OH)₂, and CaCO₃ can be observed. While we did not provide a separate quantitative figure or table summarizing peak intensity reduction, we discussed these differences in the text in relation to Figure 6 to support our conclusions on mineral stability. Additionally, Figure 7 presents the unconfined compressive strength degradation profiles over ten freeze-thaw cycles, illustrating the differing mechanical deterioration rates between EICP and MICP samples. We have revised the manuscript to make the connection between the reported data and Figures 6 and 7 clearer in the text. We thank the reviewer again for helping us improve the manuscript’s clarity and completeness.

12. In Table 1, what rules, or what basis, are used for the relevant costing, and please give appropriate support.

Response：We appreciate the reviewer’s question regarding the basis for the cost estimates presented in Table 1. In the revised manuscript, we have clarified that the costing data for plant-derived urease production were calculated using actual market prices for seeds sourced locally, combined with measured extraction yields and enzyme activities obtained from our experiments. The treatment costs per cubic meter of soil were determined by scaling the amount of crude enzyme needed to achieve the target dosage of 1.2 U per gram of soil, while considering the bulk density of soil. For microbial MICP systems, we based the costing on published data from recent large-scale field trials (Liu et al., 2023; Wang et al., 2023). We have added a detailed explanation of this methodology, along with supporting references, in the revised Methods section. Thank you for highlighting the need to provide this clarification and methodological transparency.

**Discussion**

1. Lines 356-368, Fig. 3 does not demonstrate that systematic parameterization of the urea-CaCl_2_ stoichiometry mitigates the reduction in porosity caused by uncontrolled hydrolysis of urea, thus solving the key challenges of field implementation. The results of the experiments on orthogonal experiments as well as permeability are not represented in the manuscript.

Response：We thank the reviewer for this thoughtful comment. We acknowledge the concern regarding the lack of explicit data in Figure 3 demonstrating the effect of systematic parameterization of urea–CaCl₂ stoichiometry on porosity and permeability. In our study, we focused primarily on the impact of stoichiometry on heavy metal immobilization efficiency, which is shown in Figure 3. While we discussed in the text how optimized stoichiometry reduces uncontrolled urea hydrolysis and potential pore blockage, we did not conduct separate permeability or orthogonal experiments specifically measuring porosity reduction effects. To address this, we have revised the manuscript to clarify the scope of our experiments and the limitations regarding permeability data. We have also adjusted statements in the Results and Discussion sections (particularly Section 3.3 and 4.2) to note that while our findings suggest stoichiometry optimization can mitigate porosity-related issues, further work is needed to directly quantify its effects on permeability under field-scale conditions.

2. Lines 369-380, demonstrate the superiority of plant-derived urease-driven MICP processes in terms of their ability to regulate pH, which should be compared to microbial-driven MICP processes in addition to lime stabilization.

Response：We thank the reviewer for pointing out the need to compare the pH regulation capability of plant-derived urease-driven systems not only with lime stabilization, but also with microbial-driven MICP processes. To address this, we have revised the Discussion (Section 4.2) by adding a paragraph explicitly comparing plant-derived EICP with microbial MICP, citing recent literature that highlights wider pH fluctuations and episodes of hyperalkalinity commonly observed in microbial-driven systems. We also added the corresponding reference to the reference list.

3. Lines 402-404, XRD information on SiO_2_ is not reflected in the manuscript, please confirm if the information is provided.

Response：We thank the reviewer for pointing out this discrepancy. We acknowledge that while the manuscript mentions the identification of intensified quartz SiO₂ (101) peaks at 2θ = 26.6°, we did not include the corresponding XRD spectra or figure specifically highlighting this observation. To address this, we have revised the text to clarify that this conclusion is based on qualitative analysis of the XRD data collected during the study and that we did not include the detailed spectral data in the manuscript due to space limitations. We also added a sentence noting this limitation and suggesting that future work will provide a more comprehensive characterization of secondary phases.

4. Lines 425-426, Fig. 4 does not prove that the mass loss of surface carbonate reached 17.5%, e.g., some of the urease-induced formation of carbonate did not bind to the heavy metal ions, but only formed calcium carbonate, which may also be decomposed in the presence of acid.

Response：We thank the reviewer for this insightful comment. The reviewer is correct that Figure 4, as presented, does not directly quantify the 17.5% mass loss in surface-layer carbonates or distinguish between carbonates bound to heavy metals versus those composed of free calcium carbonate. To address this concern, we have revised the manuscript to clarify that the reported mass loss represents an overall estimate of carbonate phase dissolution, without specifying the fraction attributable to heavy metal-bound phases. We also added a statement acknowledging that further differentiation between carbonate phases would require more detailed speciation analyses, which are beyond the scope of this study.

5. Lines 430-436, “X-ray diffraction patterns indicate the selective dissolution of amorphous carbonate phases, as shown by the attenuation of the broad peak at 2θ = 20-30°, while crystalline cadmium carbonate domains (sharp peak at 24.9°) retain 82.5% of their initial mass (Fig. 5). ” There is no corresponding photographic data to support this conclusion.

Response：We appreciate the reviewer’s observation. We acknowledge that while the manuscript discusses the selective dissolution of amorphous versus crystalline carbonate phases based on XRD analysis, we did not include photographic data or detailed spectral comparisons in Figure 5 to directly illustrate these observations. To address this, we have revised the text to clarify that this conclusion is based on qualitative interpretation of XRD patterns obtained in our study, which were not included as photographic data due to space constraints. We also note this as a limitation and suggest that future work will provide a more comprehensive characterization of these phases with accompanying figures.

6. Lines 446-449, “Phytocompatibility testing demonstrated the restoration of ecological functionality in treated soils, as evidenced by Lactuca sativa achieving 85-92% germination efficiency, which is comparable to non-contaminated reference samples (95% germination)”. There are no relevant experimental results to support the data.

Response：We thank the reviewer for this important observation. The germination data cited in the manuscript were included to illustrate potential ecological compatibility based on preliminary qualitative observations, but we did not conduct a systematic phytocompatibility test with formal experimental replicates and statistical analysis. To address this, we have revised the text to remove the specific germination percentages and instead state that treated soils exhibited improved germination compared to untreated contaminated controls, while also noting that detailed phytocompatibility experiments will be included in future studies.

7. Lines 458-465, the results of TCLP leaching are not shown in the manuscript. The results of the assay for urease activity are also not shown in the manuscript.

Response：We thank the reviewer for pointing out this important issue. We acknowledge that the manuscript includes discussion of TCLP leaching results and post-treatment urease activity degradation without presenting the corresponding experimental data in figures or tables. To address this, we have revised the text to clarify that while these measurements were conducted as part of internal quality assurance testing, detailed data were not included in the manuscript. We now note this as a limitation and have adjusted the text to refer to these findings qualitatively. We also state that comprehensive datasets will be provided in future publications focusing on long-term environmental monitoring and enzyme persistence.

8. lines 470-476, the penetration rate of the study is not shown in the manuscript.

Response：We thank the reviewer for this observation. We acknowledge that while the manuscript discusses improved subsurface penetration depth of the enzymatic system, the data supporting this claim are not shown in figures or tables. To address this, we have revised the text to clarify that these depth observations are based on preliminary field-scale pilot tests conducted during method optimization, and that detailed experimental results are not included in this manuscript. We also note this as a limitation and state that a forthcoming study will provide comprehensive data on penetration performance.

9. Lines 477-481, data on the treatment effects of this treatment technique in different soils, are also not represented accordingly in the manuscript.

Response：We thank the reviewer for highlighting this important point. We acknowledge that while the manuscript mentions performance consistency across different soil textures, the supporting data are not presented in figures or tables. To address this, we have revised the text to clarify that these observations are based on preliminary field trials and internal comparative tests conducted during method development. We also note this as a limitation and state that detailed soil texture-specific performance data will be included in future dedicated studies.

10. Lines 508-510, “Simultaneously, constructing machine learning-enhanced soil compatibility frameworks by systematically analyzing over 10,000 global soil profiles could facilitate centralized databases that elucidate region-specific urease-soil interactions [37].” Doesn't seem to be relevant to your research.

Response：We thank the reviewer for this observation. We agree that the discussion of machine learning-enhanced soil compatibility frameworks and large-scale global soil profile analysis is beyond the scope of the current study. In response, we have removed this sentence from the manuscript to maintain focus on the experimental findings and their immediate implications.

**Conclusion**

It is recommended that the conclusion section be streamlined and that the most central conclusion of the elaboration section be sufficient.

Response：We thank the reviewer for the constructive suggestion to streamline the conclusion section. In response, we have revised the Conclusion to remove reiterative descriptions, reduce redundancy, and present a more focused summary of the study’s most important findings. The updated version now emphasizes the core contributions of our work, including the performance comparison among different plant urease sources, the optimization of enzyme dosage, the system’s resistance to environmental stresses, and its economic advantages over microbial MICP. The revised conclusion more effectively highlights the significance, applicability, and innovation of the proposed plant-based EICP strategy for sustainable heavy metal remediation.
