# Supplementary material for: Sustainable heavy metal immobilization in contaminated soils using plant-derived urease-driven biomineralization
Source: PLoS One. 2025 Sep 19;20(9):e0331241. doi: 10.1371/journal.pone.0331241 (PMC12449025; doi:10.1371/journal.pone.0331241)
Supplement: Attachment — Submitted filename: Response_letter_auresp_2.docx [file pone.0331241.s005.docx]

**Sustainable Heavy Metal Immobilization in Contaminated Soils Using Plant-Derived Urease- Driven Biomineralization**

**Response letter to Reviewers’ comments**

**Comments from the Editors：**

Journal Requirements:

1. If the reviewer comments include a recommendation to cite specific previously published works, please review and evaluate these publications to determine whether they are relevant and should be cited. There is no requirement to cite these works unless the editor has indicated otherwise.

Response: We appreciate the opportunity to address the editorial note regarding reviewer-suggested citations. In the previous revision, we made several updates to the reference list, including the addition of eight new references, among which two ([16] and [19]) were specifically recommended by Reviewer 1. After carefully reviewing all four references suggested by Reviewer 1, we determined that [16] and [19] are highly relevant to our study and provide valuable support for our arguments. These two publications have therefore been cited appropriately in the revised manuscript. The remaining two suggested references were considered less aligned with the scope and methodology of our work, and thus were not included. Additionally, we have incorporated other recent and relevant references independently identified by the authors, to further strengthen the theoretical foundation and discussion of our manuscript.

2. Please review your reference list to ensure that it is complete and correct. If you have cited papers that have been retracted, please include the rationale for doing so in the manuscript text, or remove these references and replace them with relevant current references. Any changes to the reference list should be mentioned in the rebuttal letter that accompanies your revised manuscript. If you need to cite a retracted article, indicate the article’s retracted status in the References list and also include a citation and full reference for the retraction notice.

Response：We appreciate the editorial reminder to verify the completeness and correctness of our reference list. In this revision, we have carefully reviewed all references and made the following corrections:

[8], [16], [19]: Author names were previously inaccurate and have been corrected.

[11], [18], [37]: Missing co-authors have been added (including the use of et al. where appropriate).

[17]: The format of author initials was adjusted, and the subtitle was removed to retain only the official article title, in accordance with journal guidelines.

[36], [43]: These two references were previously cited as preprints and have now been updated with their final, formally published versions.

In addition, various minor issues related to font style, punctuation, and formatting (e.g., italicization of journal names, spacing, use of periods and commas) were corrected to fully comply with the journal's citation style. We confirm that no retracted articles were cited in the manuscript. Accordingly, there was no need to remove, replace, or annotate any retracted references.

**Additional Editor Comments :**

Please address the reference highlighted by the reviewer and revise all references throughout the manuscript.

Response：Thank you for the editorial reminder. As requested, we have addressed the reference highlighted by the reviewer by correcting the associated bibliographic information and ensuring its relevance and accuracy within the manuscript. In addition, we have comprehensively reviewed and revised all references throughout the manuscript to ensure full compliance with the journal’s formatting guidelines. Corrections included the adjustment of author names, the completion of missing co-authors, updates of preprint references to their final published versions, and formatting of journal titles, volume numbers, page ranges, DOIs, and punctuation. We believe that the reference list is now accurate, complete, and fully aligned with the journal’s requirements.

**Comments from Reviewer#2:**

Reviewer #2: The authors must fix the reference 16 (Revised version) properly. While crosschecking, it has been observed that, the author names do not match with the title. The authors must fix this issue.

Response：Thank you for pointing out the discrepancy in Reference [16]. We have carefully reviewed and corrected this reference. The author names have now been fully aligned with the article title and publication details. The updated and verified citation now reads as follows in the revised manuscript:

[16] Moghal AAB, Lateef MA, Mohammed SAS, Ahmad M, Usman ARA, Almajed A. Heavy Metal Immobilization Studies and Enhancement in Geotechnical Properties of Cohesive Soils by EICP Technique. Applied Sciences, 2020; 10(21): 7568. <https://doi.org/10.3390/app10217568>

We appreciate your attention to detail and have also re-checked all other references to ensure consistency and accuracy.
